# The Impact of Modelling Rate Heterogeneity among Sites on Phylogenetic Estimates of Intraspecific Evolutionary Rates and Timescales

**DOI:** 10.1371/journal.pone.0095722

**Published:** 2014-05-05

**Authors:** Fangzhi Jia, Nathan Lo, Simon Y. W. Ho

**Affiliations:** School of Biological Sciences, University of Sydney, Sydney, New South Wales, Australia; Field Museum of Natural History, United States of America

## Abstract

Phylogenetic analyses of DNA sequence data can provide estimates of evolutionary rates and timescales. Nearly all phylogenetic methods rely on accurate models of nucleotide substitution. A key feature of molecular evolution is the heterogeneity of substitution rates among sites, which is often modelled using a discrete gamma distribution. A widely used derivative of this is the gamma-invariable mixture model, which assumes that a proportion of sites in the sequence are completely resistant to change, while substitution rates at the remaining sites are gamma-distributed. For data sampled at the intraspecific level, however, biological assumptions involved in the invariable-sites model are commonly violated. We examined the use of these models in analyses of five intraspecific data sets. We show that using 6–10 rate categories for the discrete gamma distribution of rates among sites is sufficient to provide a good approximation of the marginal likelihood. Increasing the number of gamma rate categories did not have a substantial effect on estimates of the substitution rate or coalescence time, unless rates varied strongly among sites in a non-gamma-distributed manner. The assumption of a proportion of invariable sites provided a better approximation of the asymptotic marginal likelihood when the number of gamma categories was small, but had minimal impact on estimates of rates and coalescence times. However, the estimated proportion of invariable sites was highly susceptible to changes in the number of gamma rate categories. The concurrent use of gamma and invariable-site models for intraspecific data is not biologically meaningful and has been challenged on statistical grounds; here we have found that the assumption of a proportion of invariable sites has no obvious impact on Bayesian estimates of rates and timescales from intraspecific data.

## Introduction

In phylogenetic analyses of DNA sequence data, the evolutionary process is usually described using models of nucleotide substitution. These models commonly assume that substitutions occurring at each site are described by a Markov chain and that different sites evolve in a mutually independent manner. In practice, almost all models of nucleotide substitution are time-reversible. The general time-reversible (GTR) model, formally described by Tavaré [1], includes parameters that allow unequal frequencies for the four nucleotides and a distinct rate for each of the six pairwise nucleotide substitutions. By constraining one or more of these parameters, a family of time-reversible models can be generated. This gives rise to special cases of the GTR model, such as the Jukes–Cantor [2], Kimura [3], and Hasegawa–Kishino–Yano [4] models.

In their basic form, nucleotide substitution models assume that the evolutionary process is homogeneous across sites. In reality, however, rates of mutation can vary among sites because of selective pressures associated with structural and functional constraints [5]–[7]. Some sites, such as CpG islands in mammalian taxa, have a higher propensity to mutate [8]. Failure to take into account this rate heterogeneity among sites (RHAS) can lead to biased estimation of branch lengths, with corresponding impacts on estimates of phylogenies, substitution rates, and evolutionary timescales [9]–[13].

RHAS can be modelled in a number of ways, but the most popular approach is to assume that the rate at each site is a random variable drawn from a statistical distribution. The gamma distribution (+Γ) is most commonly used for this purpose [14], [15], owing to its ease of interpretation and its good fit to empirical data [5]. The shape of the gamma distribution, governed by the shape parameter α, can range from bell-shaped (α>1) to L-shaped (α<1). Consequently, the gamma distribution is capable of modelling various degrees of RHAS [5].

To reduce computational burden, most methods employ a discrete gamma model in which the continuous distribution is approximated by several rate classes with equal percentiles and probabilities [14]. Within each class, all of the rates are represented by the mean or median. The higher the number of rate categories, the better is the fit of the discrete gamma distribution to the continuous gamma distribution. The number of rate categories (*k*) used in phylogenetic analyses of nucleotide sequences generally ranges from 2 to 32 [16]–[18], with most analyses employing 4–10 categories. In an analysis of two small data sets (4 sequences, 1352 sites and 5 sequences, 570 sites), Yang [14] found that 4 rate categories provided sufficiently good approximations of α and the likelihood and that there was little improvement in estimation accuracy when more than 8 categories were used. A greater number of rate categories might be preferable for large data sets [19].

RHAS can also be described using discrete rate-class models, in which the rate categories are not determined from some underlying distribution. A special case of the discrete-rates model is the invariable-sites model (+I), which assumes that there is a rate class with a rate of 0 [4], [20]–[23]. In this model, some proportion of sites (*p*_inv_) is assumed to be invariable, or completely resistant to change, because mutations at these sites are strongly deleterious or fatal. The remaining sites are assumed to evolve at non-zero rates. The idea of invariable sites was inspired by studies of protein structures and has intuitive appeal [20], [24]. The invariable-sites model can be combined with the gamma model of RHAS (+Γ+I) [25], [26], and the resulting gamma-invariable mixture models are widely used.

When performing a phylogenetic analysis, the inclusion of a model of RHAS is usually determined using a model-selection approach. A common practice is to compare a range of substitution models using a model-selection criterion, such as the Akaike information criterion [27] or Bayesian information criterion [28]. In this respect, the Bayesian information criterion has been shown to perform well across a range of simulation scenarios [29]. The best-fitting model is then used in subsequent analyses of the data set.

Evolutionary models chosen using objective criteria are not always the most biologically pertinent [30], which raises questions about the meaning of the resulting estimates of parameters. A prominent example is the +I model, which is sometimes selected as the best-fitting RHAS model for data that have been sampled at the population level. Intraspecific data tend to display lower levels of variation than sequences that have been sampled at the species level and above. This makes the evaluation of *p*_inv_ highly sensitive to taxon sampling [5]. We would expect the number of constant sites to decline as sample size increases, leading to lower estimates of *p*_inv_. Sites observed to be constant among sequences might not be invariable, but might simply have not experienced any mutations among the sequences that have been sampled. Additionally, deleterious mutations are much more likely to be found in population-level data [31], [32], so that sites that would typically be treated as ‘invariable’ at the phylogenetic level might contain transient polymorphisms at the population level.

The +Γ+I mixture model, first used by Gu *et al.* [25], has been criticised on the grounds that the two parameters involved – *p*_inv_ and α – cannot be optimised independently of each other [15], [19], [33], [34]. An L-shaped gamma distribution (α<1) already accommodates a proportion of low-variability sites; as a consequence, adding a parameter to account for invariable sites creates a strong correlation between *p*_inv_ and α [34]. This might cause considerable problems during the parameter optimisation process, since it is impossible to obtain reliable estimates of both parameters simultaneously [33]. Combining this with the aforementioned sensitivity of *p*_inv_ to the size of the data set and the level of divergence, applying the +Γ+I model to intraspecific data sets appears particularly problematic, at least on theoretical grounds.

The impact of using different RHAS models in phylogenetic analyses of intraspecific data is not well understood. The impact of the choice of RHAS model is most likely to be seen in estimates of branch lengths, which can have subsequent effects on the inferred tree topology. Here we investigate how varying the RHAS model affects phylogenetic analyses based on the molecular clock. We focus on five intraspecific data sets, four of which comprise heterochronous sequences (ancient DNA and viruses). In heterochronous data set, the ages of the sequences provide internal calibrations for the molecular clock, making such data ideal for studying evolutionary rates and timescales at the intraspecific level. We test whether increasing the number of gamma categories or assuming a proportion of invariable sites affects estimates of substitution rates and coalescence times.

## Materials and Methods

We assembled five intraspecific data sets: (i) complete mitogenomes from human haplogroup C1, (ii) complete mitogenomes from hominins, (iii) mitochondrial D-loop from muskox, (iv) *PB2* gene from H1N1 human influenza virus, and (v) concatenated genome fragment from HIV-1. The first data set comprised isochronous sequences from the present day, whereas the last four data sets comprised heterochronous sequences of known ages. Details of the datasets are listed in Table 1.

**Table 1:** Details of five intraspecific data sets analysed in this study.

Species	Marker	Length (bp)	Sequences (ancient/modern)	Temporal span (years)	Best-fitting model (BIC)	Molecular clock	Primary source
Human (hg C1)	Mitogenome	15,031	0/184	–	TrN+I+Γ	Strict	–
Hominin^a^	Mitogenome	16,607	61/56	65000	TrN+I+Γ	Strict	[50]–[52]
Muskox (*Ovibos moschatus*)	D-loop	682	104/16	45740	TVM+I+Γ	Uncorrelated lognormal	[53]
Human influenza A H1N1 virus	*PB2*	2345	115/16	82	TVM+Γ	Uncorrelated lognormal	–
HIV-1	Concatenated genome fragments	1063	157/4	46	GTR+I+Γ	Uncorrelated lognormal	[54]

a The hominin data set includes sequences from 56 extant and 55 ancient modern humans (*Homo sapiens*), 5 Neanderthals (*Homo neanderthalensis*), and the Denisovan hominin.

For each data set, sequence alignments were done using MUSCLE 3.8.31 [35] and adjusted manually. Sequences with uncertain ages were removed. For each data set, we performed preliminary Bayesian phylogenetic analyses using the best-fitting substitution models selected by the Bayesian information criterion. Alignments for all datasets used in this study are available in File S1. Bayesian phylogenetic analyses of the data sets were performed in BEAST v1.7.5 [36]. The best-fitting model of nucleotide substitution was selected for each data set using the Bayesian information criterion in Modelgenerator 0.85 [30]. We tested four different RHAS models: equal rates among sites, +Γ, +I, and +Γ+I. For the +Γ models, we repeated the analysis using various numbers of rate categories ranging from 3–32. To test for rate heterogeneity among lineages, we initially used the uncorrelated lognormal relaxed clock [37]. For the human mitogenome data sets (i and ii), the coefficient of rate variation did not provide any evidence of rate variation among lineages. Marginal likelihoods were estimated using the harmonic-mean estimator [38].

Using the maximum-clade-credibility trees from our Bayesian analyses, we inferred the number of substitutions at each site using stochastic mutational mapping in SIMMAP [39], [40]. We used the empirical nucleotide frequencies and applied the mean rate estimate from the Bayesian phylogenetic analysis of each data set to scale the branch lengths. Numbers were rounded to the nearest integer. The distribution of inferred mutational counts provided a picture of the RHAS pattern for each data set. We performed chi-squared tests to compare the goodness-of-fit of gamma and negative binomial distributions to the site-specific substitution counts. In the absence of RHAS, the distribution of site-specific substitution counts should conform to the Poisson distribution. In the presence of gamma-distributed RHAS, we expect the number of changes per site to conform to the negative binomial distribution. We simultaneously estimated the values of α by minimising χ^2^. We compared the fit of these two distributions using the Akaike information criterion.

In the analyses of the heterochronous sequences (data sets ii to v), the molecular clock was calibrated using the ages of the sequences. Following previous studies of viruses [41] and ancient DNA [42], we used date-randomisation tests to check that the spread and structure of the sequence ages were sufficient for calibrating estimates of substitution rates (Figure S1). This test involves the random reassignment of the dates to the sequences. If the mean posterior rate estimated from the original data set is included in any of the 95% credibility intervals of the rates estimated from the date-randomised replicates, the sequence ages are considered to have insufficient structure and spread for calibrating the molecular clock. For the mitogenomes from human haplogroup C1 (data set i), we assumed an age of 21,700 years (standard deviation 2700 years) for the root of the tree, following the study by Kumar *et al.* [43].

A constant-size coalescent prior was used for each analysis, based on Bayes factors calculated from the marginal likelihoods of each +Γ model. Posterior distributions of parameters, including the tree, were estimated by Markov chain Monte Carlo (MCMC) sampling. MCMC chains were run for at least 2×10^7^ steps, with samples drawn every 10^3^ steps. After discarding an appropriate proportion of the samples as burn-in, we checked for acceptable sampling, mixing, and convergence to the stationary distribution in each case.

## Results

### Analysis of rate variation among sites

**Figure 1:**
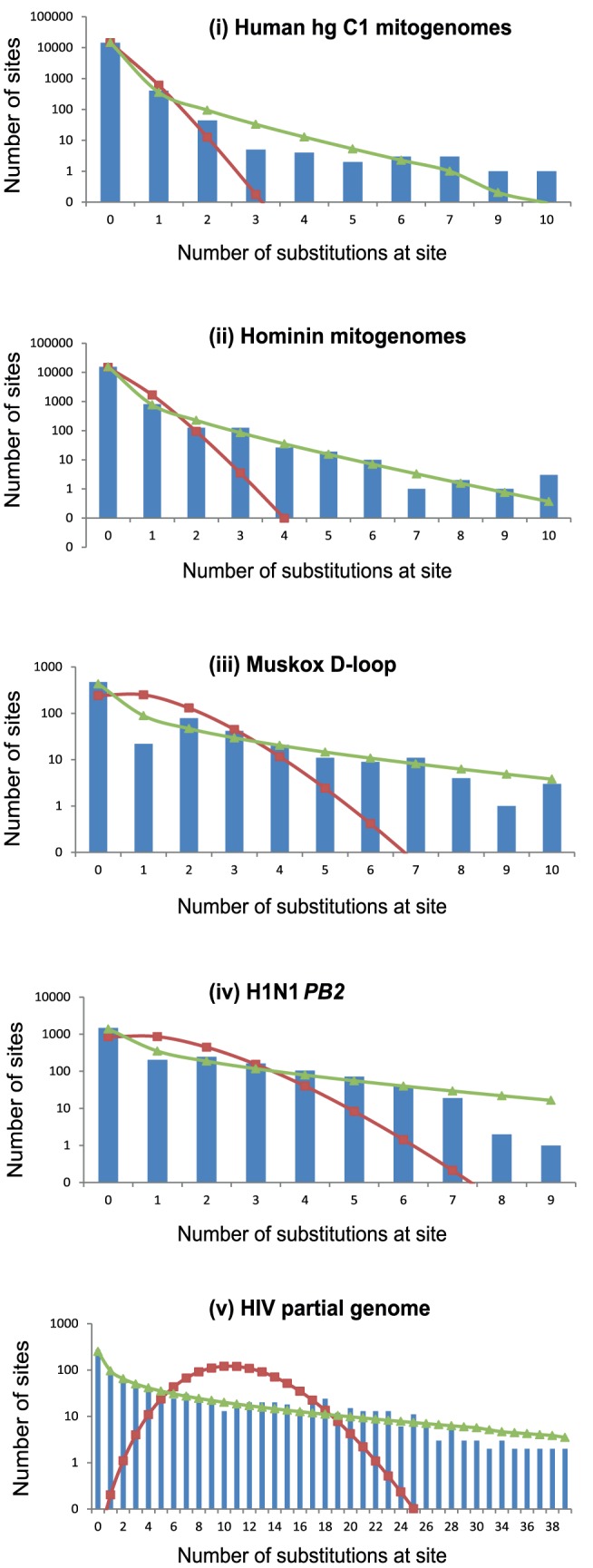
Semi-logarithmic plots of substitutions per site for five intraspecific DNA data sets. Number of substitutions at each site were inferred using parsimony on the Bayesian estimates of the tree topologies. Columns indicate the number of sites against the number of substitutions occurring at each site. Red and green lines indicate the best-fitting Poisson and negative binomial distributions, respectively.

All data sets showed a negative relationship between the number of sites in a rate category and the number of inferred substitutions per site. The category of zero inferred changes had the highest count for all data sets and, with the exception of the HIV data set, most sites were observed to be constant for each data set (Figure 1). For all data sets, the Poisson distribution provided a poor fit to the site-specific substitution counts (P<<0.001), with the negative binomial distribution providing a significantly better fit (Table 2). Despite this improvement, neither model provided a good approximation of the pattern of RHAS in the two human mitogenome data sets (i and ii), which were the largest and least variable. The HIV data set (v) features a large proportion of sites inferred to be non-constant, and shows considerable deviation from both the Poisson and negative binomial distributions. There was a particularly large proportion of sites with high inferred numbers of substitutions.

**Table 2:** Fit of Poisson and negative binomial distributions to the site-specific substitution counts in Figure? 1, estimates of α, the ratio of the P values for both distributions, the proportion of constant sites for five data sets and the substitution rate estimate for each data set.

	Poisson	Negative binomial	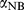 a	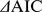	Number of constant sites^b^	Mean substitution rate (site^−1^ year^−1^)
**(i) Human hg C1 mitogenomes**	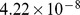	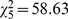	0.048	809.7	96.8%	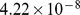
	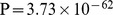	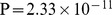				
**(ii) Hominin mitogenomes**	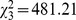	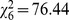	0.092	2617.6	93.2%	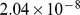
	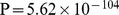	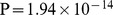				
**(iii) Muskox**	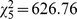	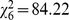	0.24	829.0	69.5%	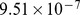
	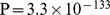	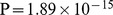				
**(iv) H1N1**	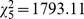	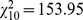	0.31	1622.0	63.6%	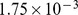
	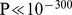	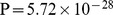				
**(v) HIV**	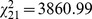	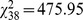	0.39	13845.0	27.5%	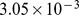
	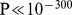	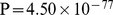				

a This estimate of the shape parameter for gamma-distributed rates among sites was obtained by minimising χ^2^.

b Sites having <0.5 mutations as inferred by stochastic mutational mapping in SIMMAP.

### Effect of RHAS model on estimates of likelihoods and parameters

**Figure 2:**
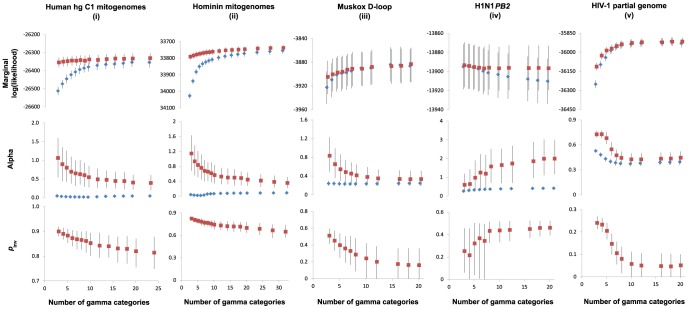
Bayesian phylogenetic estimates of various parameters against number of gamma rate categories for five intraspecific DNA data sets. From top to bottom, rows show estimates of marginal likelihood, the gamma shape parameter (α), and the proportion of invariable sites (*p*_inv_). Filled blue and empty red markers represent parameter estimates using +Γ and +Γ+I models, respectively.

**Figure 3:**
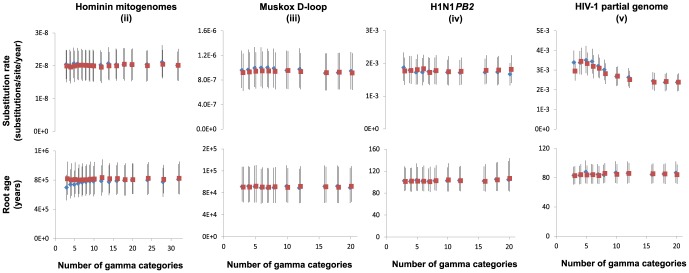
Bayesian phylogenetic estimates of substitution rate and root age against number of gamma rate categories for four intraspecific DNA data sets. Filled blue and empty red markers represent parameter estimates using +Γ and +Γ+I models, respectively.

For four out of the five data sets, the marginal likelihood increased with the number of gamma rate categories (Figure 2). In general, 6–10 rate categories provided a good approximation of the asymptotic likelihood value for +Γ models, whereas there was minimal improvement in likelihood when using greater than 10 gamma rate categories. It is noticeable that marginal likelihood is generally higher with +Γ+I models than with +Γ models, and that there is more rapid convergence towards the asymptotic value in +Γ+I models. In the analyses of the four heterochronous data sets (ii–v), neither varying the number of gamma rate categories nor allowing a proportion of invariable sites had any obvious impact on estimates of the coalescence time (root age) or the substitution rate (Figure 3). In almost all cases, the 95% credibility intervals overlapped substantially and the variance in means was <6% of the average 95% CI width (except for the HIV data set). The only exception was the rate estimate for the HIV data set (v), which slightly decreased with an increasing number of gamma rate categories. In the analysis of the mitogenome sequences from human haplogroups C1 (data set i), neither varying the number of gamma categories nor allowing a proportion of invariable sites had any noticeable effect on the estimate of the substitution rate (Figure S2).

The relationships between the α shape parameter or *p*_inv_ and the number of gamma rate categories were less clear (Figure 2). The general pattern seemed to be a negative relationship between both parameters and the number of gamma rate categories, which can best seen in the declining posterior means and the non-overlapping 95% CIs of estimates based on small and large numbers of gamma categories. However, exceptions to both of these rules were observed in the sequences from human influenza A H1N1 virus (data set iv).

**Figure 4:**
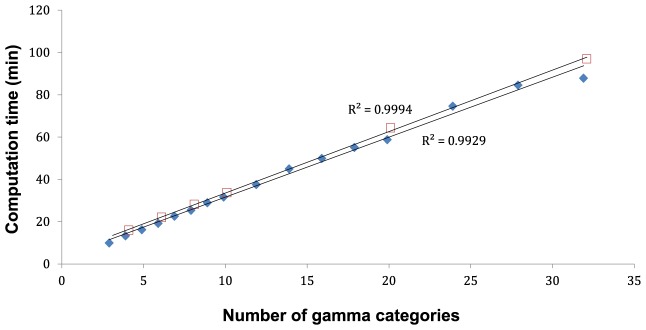
Computation time (min) as a function of the number of gamma rate categories, for the hominin mitogenome data set (ii). Filled blue and empty red markers represent computation time using TrN+Γ and TrN+I+Γ models, respectively. Each Markov chain was run for 10^6^ steps on a six-core processor (Intel Xeon W3690).

Increasing the number of gamma rate categories led to a linear increase in computation time (Figure 4). The computational costs associated with an increase in the number of gamma categories were similar between +Γ and +Γ+I models. With regard to the topologies of the maximum-clade-credibility trees, we observed that the major clades were unaffected by changes in the number of gamma categories or the inclusion of invariable sites (results not shown). Since this study is limited to data at the population level, which are described by the coalescent process, we did not investigate the impacts of different models on detailed phylogenetic relationships.

## Discussion

Our phylogenetic analyses of five intraspecific data sets have provided a number of insights into the performance of RHAS models. Varying the RHAS model, including the number of gamma rate categories or a proportion of invariable sites (*p*_inv_), had negligible impacts on our estimates of root age and substitution rate from heterochronous data sets. We found evidence of a complex interplay between α, the number of gamma rate categories, and *p*_inv_. When a proportion of the sites were assumed to be invariable, increasing the number of gamma rate categories generally caused a decrease in both α and *p*_inv_. This is because the sites that are changing rapidly (mutational hotspots) are preferentially accommodated over less variable sites when there are few rate categories. When there is a limited number of rate categories, this results in a gamma distribution with a higher α. The presence of the invariable-sites parameter in this situation mitigates this bias, but results in an overestimation of *p*_inv_.

Our results highlight a trade-off between computational cost and improved accuracy when an increasing number of gamma rate categories are used to model RHAS. The marginal likelihood, which describes the average fit of a model to the data, reaches a plateau as the number of gamma rate categories increases, a result that echoes those of Yang [14]. Using a large number of categories, however, incurs a significant computational cost, increasing both the RAM and time requirements for the likelihood calculations [44]. Our results suggest that using 6–10 rate categories provides a good approximation of the plateau likelihood value when not using invariable-sites models, and that increasing the number of rate categories incurs greater computational cost with minimal benefit. This contradicts suggestions that using a relatively small number of rate categories is insufficient to capture the complexities of the molecular evolutionary process [17].

For population-level analyses that aim to estimate substitution rates or coalescence times, our results suggest that greatly increasing the number of gamma categories typically does not lead to substantial changes in parameter estimates. An exception is when the evolutionary rate is highly variable among sites and deviates strongly from a gamma distribution, as in the case of the HIV data set examined here. In such cases, a higher number of rate categories (8–10) might lead to a modest improvement in estimation accuracy. Surprisingly, the H1N1 influenza virus data did not show a positive relationship between marginal likelihood and the number of rate categories, although the reasons for this are unclear.

The results of our analyses using the invariable-sites model are more complex. The +Γ+I model is intuitively appealing and is, according to the Bayesian information criterion, the preferred model for four of the five data sets analysed (Table 1). However, estimates of parameters in the +Γ+I model are highly sensitive to taxon sampling [5], [34] and there is a strong correlation between the proportion of invariable sites and the gamma shape parameter [34]. Our analyses reveal that estimates of the proportion of invariable sites are also highly susceptible to changes in the number of rate categories. Our results corroborate the notion that estimates of both parameters are inevitably biased when the +Γ+I model is used [34].

The interdependence of *p*_inv_ and α in the +Γ+I model has led some researchers to warn against its use [33], [45]. For example, in the justification of their exclusion of +Γ+I models in their analyses, Ren *et al.* [45] contended that one should also consider the “biological interpretations of the models and the robustness of analysis to model assumptions” (p. 815), not just the fit of the model to the data. We concur with this viewpoint, considering that the invariable-sites assumption is particularly troublesome for intraspecific data. Here we would expect a +Γ+I model to perform well for data sets that show a bimodal distribution in site-specific rates (one peak at 0 and one peak at >0). However, when the aim is to estimate the substitution rate or coalescence times for a population-level data set, we found that allowing a proportion of invariable sites did not alter the results substantially. There was, however, a small computational benefit associated with +Γ+I models, since they generally outperformed +Γ models in marginal likelihood at small numbers of gamma categories, reducing the need for higher numbers of categories in +Γ models. In some instances (data set i and ii), the asymptotic marginal likelihood for +Γ+I models was slightly higher than the marginal likelihood for +Γ models.

Using a gamma distribution to model RHAS has deservedly been popular, owing to its good fit and mathematical simplicity [46]. There is, however, no reason to believe that the distribution of rates among sites actually follows the gamma distribution. Indeed, we observe that the gamma model still does not provide an accurate picture of RHAS, especially for the least variable data sets that we examined (i and ii). Some studies have explored the possibility of using alternative approaches to model RHAS. Notably, the discrete-rates CAT model, implemented in RAxML [47] and FastTree2 [44], has been shown to be computationally more efficient than the traditional +Γ model and yields tree topologies with improved likelihood values [48]. Recently, Wu *et al.* [49] proposed a Bayesian method of automatic model selection that simultaneously estimates the substitution model and rate at each site. The performance of these parameter-rich models, with regard to phylogenetic analyses of intraspecific sequence data, warrants further study.

## Conclusions

The inclusion of a parameter for the proportion of invariable sites is a legacy of studies conducted at the interspecific level. At the intraspecific level, estimating the proportion of invariable sites is primarily a statistical measure that does not have much biological meaning. Here we suggest that most intraspecific studies of substitution rates or coalescence times can use fewer than 10 gamma rate categories, in order to achieve a balance between model complexity, computational efficiency, and parameter estimation. The results of our study apply to population-level data, but are probably relevant to data sets containing sequences from closely related species. Further studies of rate variation in interspecific data sets will provide additional insights into the performance of RHAS models.

## Supporting Information

Figure S1 **Results of the date-randomisation test for temporal signal in heterochronous data.** The plot shows the rate estimates and their 95% credibility intervals for the unrandomised data (empty markers)_and date-randomised replicates (filled markers). The test was conducted on the four heterochronous data sets, each with 20 replicates.
(EPS)

Figure S2 **Estimates of substitution rates as a function of the number of gamma rate categories, for the isochronous human hg C1 mitogenome data set (i).** Filled blue and empty red markers represent rate estimates using +Γ and +Γ+I models, respectively.
(EPS)

File S1 **Alignments for all datasets used in this study.**
(ZIP)
